# Global experience and progress in GreenLight-XPS 180-Watt photoselective vaporization of the prostate

**DOI:** 10.1007/s00345-022-03997-2

**Published:** 2022-05-02

**Authors:** Isabel Lichy, Kyle Law, Côme Tholomier, David-Dan Nguyen, Iman Sadri, David Bouhadana, Félix Couture, Ahmed S. Zakaria, Naeem Bhojani, Kevin C. Zorn, Franck Bruyère, Luca Cindolo, Giovanni Ferrari, Carlos Vasquez-Lastra, Tiago J. Borelli-Bovo, Edgardo F. Becher, Vincent Misrai, Dean Elterman, Maximilian Reimann, Hannes Cash

**Affiliations:** 1 Department of Urology, Charité–University Medicine Berlin, Hindenburgdamm 30, 12203 Berlin, Germany; 2 Prouro, Urology Berlin, Berlin, Germany; 3 Department of Urology, University of Magdeburg, Magdeburg, Germany; 4 Department of Urology, University of Montreal Hospital Center (CHUM), Montreal, Canada; 5 Faculty of Medicine and Health Sciences, McGill University, Montreal, Canada; 6 Division of Urology, Department of Surgery, McGill University, Montreal, QC Canada; 7 Division of Urology, Department of Surgery, Northern Ontario School of Medicine, Thunder Bay, ON Canada; 8 Department of Urology, University Hospital Magdeburg, Magdeburg, Germany; 9 Department of Oncology and Urology, Centre Hospitalier Universitaire de Tours, Centre-Val de Loire, France; 10 Department of Urology, Hesperia Hospital, Cure Group, Modena, Italy; 11 Department of Urology, ABC Medical Center, Mexico City, Mexico; 12 Borelli Urologia, Ribeirão Presto, Brazil; 13 Centro de Urologia, CDU, Buenos Aires, Argentina; 14 Department of Urology, Clinique Pasteur, Toulouse, Midi-Pyrenees France; 15 Division of Urology, Dept. of Surgery, University Health Network, University of Toronto, Toronto, ON Canada

**Keywords:** Global experience, Greenlight, BPH, GL-XPS, Global Greenlight Group

## Abstract

**Purpose:**

To evaluate changes in global perioperative data of GreenLight-XPS 180-Watt photo-selective vaporization of the prostate (GL-XPS) of the Global Greenlight Group (GGG) database.

**Methods:**

3441 men, who underwent GL-XPS for symptomatic BPH between 2011 and 2019 at seven high volume international centers, were included. Primary outcome measurements were operative time (OT; min), effective laser time (LT; min of OT), as well as intraoperative and postoperative adverse events (AEs), all analyzed by year of surgery (2011–2019) and prostate volume (PV) group (< 80 ml vs. 80-150 ml vs. > 150 ml).

**Results:**

The median age was 70 years (interquartile range 64–77), the median PV was 64 ml (IQR 47–90). The OT and LT slightly increased but stayed highly efficient all in all. Median OT was 60 min (IQR 45–83) and LT was 33 min (IQR 23–46). Median energy use was 253 kJ (IQR 170–375) with an energy density of 3.94 kJ/ml (IQR 2.94–5.02). The relative probability of perioperative AEs decreased by 17% each year (*p* < 0.001). The relative probability of perioperative transfusion dropped significantly from 2% in 2011 to 0% in 2019 (*p* = 0.007). The early postoperative complications (within 30 days after surgery) decreased significantly from 48.8% (*n* = 106) in 2011 to 24.7% (*n* = 20) in 2019 (*p* > 0.001).

**Conclusion:**

These findings from the GGG demonstrate significant improvement secondary to growing experience with GL-XPS between 2011 and 2019 in intraoperative AEs, including transfusions, and postoperative AEs. While staying highly efficient in OT and LT of GL-XPS within a 9-year period of experience.

## Introduction

Following the failure of conservative medical management to adequately address lower urinary tract symptoms (LUTS) and elevated post-void urine volume secondary to benign prostate hyperplasia (BPH), surgical therapy is often recommended. Transurethral resection of the prostate (TURP) remains the current surgical gold standard for prostates between 30 and 80 ml [1] but this procedure is associated with several perioperative adverse events (AEs) such as bleeding [2], especially in larger prostates [3]. GreenLight-XPS 180-Watt photoselective vaporization of the prostate (GL-XPS) has been added to the international guidelines as a safe surgical method with decreased morbidity especially in prostates > 100 ml and for patients requiring therapeutic anticoagulation therapy [1–5].

In 2014, the GOLIATH study confirmed non-inferiority of GL-XPS compared to TURP regarding IPSS-QoL, Qmax, and AEs [6]. Since then, with growing global experience, the surgical technique and application of GL-XPS have certainly evolved. We have previously shown that growing experience with GL-XPS at a single high-volume center is associated with improved OT and LT over a 5-year period [7]. However, there are currently no studies that have evaluated the global progress of GL-XPS in respect to effectiveness, efficacy, and safety.

As such, this study sought to evaluate the perioperative progress of GL-XPS secondary to growing global experience using data from the Global Greenlight Group (GGG) database which represents multiple, international high-volume centers. The primary outcomes were operative and laser time (OT, LT; min), as well as intraoperative and postoperative AEs, all analyzed by prostate volume (PV) (ml) and by the year of surgery.

## Patients and methods

### Patients and study design

**Fig. 1:**
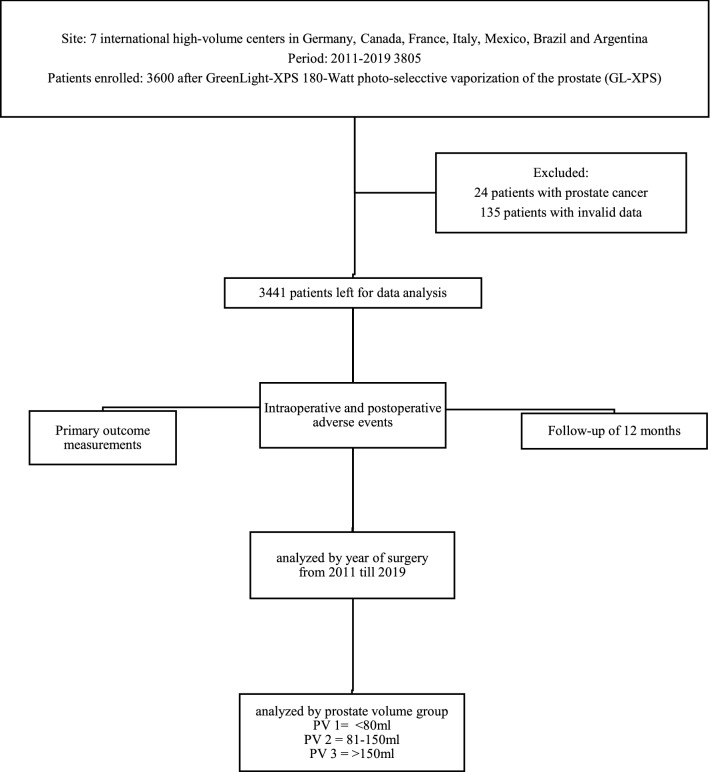
Clinical research flowchart

This retrospective analysis of the GGG database included 3441 men who underwent GL-XPS (Boston Scientific, Minnetonka, MN, USA) for symptomatic BPH between 2011 and 2019 from one of eight experienced surgeons at seven international high-volume centers in Germany, Canada, France, Italy, Mexico, Brazil, and Argentina [8]. Our study design was visualized in Fig. 1. High Data quality/density was ensured using objective variables and a close cooperation between the individual centers of the Global Greenlight Group.

### Study outcomes

Primary outcome measurements were OT, LT, perioperative AEs, and perioperative transfusions analyzed by year of surgery (2011–2019) and by PV (PV1: < 80 ml vs. PV2: 80–150 ml vs. PV3: > 150 ml) with respect to effectiveness, efficacy, and safety with a postoperative follow-up of 12 months.

Postoperative follow-up included International Prostate Symptom Score–Quality of Life (IPSS–QoL), early postoperative AE (< 30 days, subcategorized in low-grade (Clavien-Dindo grade I–II) and high-grade (need of an intervention; Clavien-Dindo grade > IIIa–IV), hospital length of stay (days), catheterization time (days), and postoperative re-catheterization rate.

### Surgical procedures

All eight GL-XPS surgeons were fellowship-trained urologists equally experienced in performing GL-XPS regularly. GL-XPS was performed using the XPS-180 W system and MoXy™ laser fiber. Procedures were performed in accordance to published international guidelines [2, 9]. Procedures were performed under general or spinal anesthesia. Prophylactic antibiotic treatment, as well as antithrombotic therapy were administered according to local guidelines.

### Statistical analysis

Non-normally distributed data are presented as a median (IQR) or as a percentage. Categorical variables were presented by relative frequencies. Linear regression analysis and logistic regression analysis were conducted for the data set to visualize the trend of the years of surgery. Multivariable logistic regression analysis was performed to detect influencing factors. Statistical significance was set at a two-sided *p* < 0.05. Data were analyzed with SPSS, version 27 (Chicago, Illinois, USA).

## Results

### Preoperative baseline results

Patient baseline characteristics of the 3441 men are summarized in Table 1. The median age was 70 years (interquartile range IQR 64–77) with a median PV of 64 ml (IQR 47–90) and a median PSA level of 3.27 ng/dl (IQR 1.76–5.88). Between 2011 and 2019, there was a significant increase in PV treated (*p* = 0.02) by 0.71 ml each year. In 2011, there were 28.7% of patients with PV2, 3% PV3 and in 2019 it raised to 43.6% in PV2 and 4% in PV3. The median IPSS/QoL was 23/4 (IQR 19–27/3–5).

**Table 1:** Patients demographics between 2011 and 2019 (linear regression or binaer logistic regression)

*n* = max. 3441	Baseline	2011		2012		2013		2014	
	Median/*n* (IQR/%)	Median/*n*	IQR/%	Median/*n*	IQR/%	Median/*n*	IQR/%	Median/*n*	IQR/%
Age, years	70 (64–77)	70	65–87	71	64–77	70	63–77	70	64–76
PSA, ng/dl	3.27 (1.76–5.88)	3.76	2–7	2.84	1.60–5.48	3.55	2–5.6	3.2	1.9–5.4
TRUS volume, ml	64 (47–90)	61	49–89	62.00	43–88	63	45–83	63	48–90
IPSS baseline	23 (19–27)	23	19–27	22.50	18–29	22	19–28	23	19–27
QoL baseline	4 (3–5)	5	4–5	5.00	4–6	5	4–5	5	4–5
*Q*_max_ baseline, ml/s	6 (4–8)	6	4–8	5.5	3–7	6	4–7	5.5	4–8
PVR, ml	120 (30–273)	175	80–418	151.5	66–400	168	65,5–314	126.5	50–333
Medication use	729 (60.6%)	75	52.8%	101	54.6%	88	53.7%	88	55.7%
Anticoagulation use	1176 (35%)	87	39.7%	115	33.8%	113	31.6%	173	34.7%
α-blocker use	2661 (79.9%)	182	83.9%	280	83.3%	288	81.4%	390	80.1%
5α-reductase inhibitor use	1424 (42.6%)	118	54.1%	179	53.1%	159	44.9%	176	36.1%
Median Lobe	532 (36.2%)	54	36.2%	77	36.0%	57	26.4%	57	29.2%
ASA	428 (22%)	28	18.9%	59	25.4%	37	16.5%	32	16.9%
	996 (51.2%)	83	56.1%	108	46.6%	106	47.3%	88	46.6%
	504 (25.9%)	35	23.6%	62	26.7%	78	34.8%	66	34.9%
	18 (0.9%)	2	1.4%	3	1.3%	3	1.3%	3	1.6%
Retention, preoperative	342 (35.8%)	39	47.6%	45	36.3%	53	42.4%	54	43.9%
Foley in place, preoperative	841 (27.7%)	69	31.4%	90	28.6%	94	29.8%	104	24.8%

*n* = max. 3441	Baseline	2015		2016		2017		2018		2019	
	Median/*n* (IQR/%)	Median/n	IQR/%	Median/*n*	IQR/%	Median/*n*	IQR/%	Median/*n*	IQR/%	Median/*n*	IQR/%
Age, years	70 (64–77)	69	64–76	70	64–76	71	65–77	70	64–77	70.5	64–77
PSA, ng/dl	3.27 (1.76–5.88)	3.2	1.7–5.2	3	1.52–5.76	3.15	1.62–5.7	3.61	1.91–7.33	4.5	2.39–7
TRUS volume, ml	64 (47–90)	65	47–88	61	46–85	64.5	47–90	70.5	50–96	78	51–108
IPSS baseline	23 (19–27)	23	19–27	22	19–27	22	19–27	22	17–27	24	19–30
QoL baseline	4 (3–5)	5	4–5	4	3–5	3	0–5	4	2–5	5	5–6
*Q*_max_ baseline, ml/s	6 (4–8)	6	4–9	6	4–8	6	4–9	6	4–8	6	4–7
PVR, ml	120 (30–273)	112.5	20–230	100	0–234	99.5	0–200	67.5	0–191.5	134.5	66.5–347
Medication use	729 (60.6%)	71	58.7%	74	63.8%	78	75.0%	86	78.9%	68	66.0%
Anticoagulation use	1176 (35%)	194	34.5%	219	36.9%	129	35.1%	115	36.2%	31	29.0%
α-blocker use	2661 (79.9%)	429	77.3%	455	76.9%	304	84.0%	240	75.2%	93	86.1%
5α-reductase inhibitor use	1424 (42.6%)	209	37.6%	219	37.0%	173	47.0%	133	41.7%	58	53.7%
Median Lobe	532 (36.2%)	59	35.8%	48	29.4%	56	37.6%	60	51.3%	64	64.0%
ASA	428 (22%)	56	17.8%	86	20.9%	55	29.4%	51	36.4%	24	24.0%
	996 (51.2%)	174	55.2%	224	54.5%	94	50.3%	71	50.7%	48	48.0%
	504 (25.9%)	84	26.7%	98	23.8%	36	19.3%	18	12.9%	27	27.0%
	18 (0.9%)	1	0.3%	3	0.7%	2	1.1%	0	0.0%	1	1.0%
Retention, preoperative	342 (35.8%)	35	32.4%	42	35.3%	30	25.2%	24	26.7%	20	31.3%
Foley in place, preoperative	841 (27.7%)	129	25.4%	138	25.7%	104	31.8%	96	31.8%	17	18.5%

*n* = max. 3441	Baseline	*F*	Coefficient variable	Coefficient constant	*R*^2^	adjusted *R*^2^	*p*
	Median/*n* (IQR/%)						
Age, years	70 (64–77)						0.778
PSA, ng/dl	3.27 (1.76–5.88)						0.350
TRUS volume, ml	64 (47–90)	*F* (df = 1;3245) = 5.38*	0.711*	− 1360.733*	0.002	0.001	**0.020**
IPSS baseline	23 (19–27)						0.118
QoL baseline	4 (3–5)	*F* (df = 1;952) = 69.443***	− 0.188***	383.118***	0.068	0.067	** < 0.001**
*Q*_max_ baseline, ml/s	6 (4–8)						0.516
PVR, ml	120 (30–273)	*F* (df = 1;1837) = 68.053***	− 15.84***	32,102.142***	0.036	0.035	** < 0.001**
Medication use	729 (60.6%)		1.134***	0.00***	0.031		** < 0.001**
Anticoagulation use	1176 (35%)						0.907
α-blocker use	2661 (79.9%)						0.078
5α-reductase inhibitor use	1424 (42.6%)		0.959*	**1.583 × 10**^6^*	0.003		**0.012**
Median Lobe	532 (36.2%)		1.116***	0.00***	0.022		** < 0.001**
ASA	428 (22%)						
	996 (51.2%)						
	504 (25.9%)						
	18 (0.9%)						
Retention, preoperative	342 (35.8%)		0.901***	**1.042 × 10**^6^***	0.019		** < 0.001**
Foley in place, preoperative	841 (27.7%)						0.668

Bold means the significans with *p* < 0.05

*TRUS* transrectal ultrasound, *PSA* prostate-specific antigen, *IPSS* International Prostate Symptom Score, *QoL* Quality of Life, *Q*_max_  maximum flow rate, *PVR* post void residual volume, *OT* operation time; *LT* effective laser time, *Energy density* energy used per TRUS volume, *ASA* American Society of Anesthesiologists—classification results are represented as median (IQR = interquartile range) or results are represented as *n* = count and relative percentage

*p < 0.05, **p < 0.01, ***p < 0.001

The median post-void residual volume was 120 ml (IQR 30–273) and showed a significant decrease between the years of surgery (*p* < 0.001). Additionally, there was a significant increase in patients with a median lobe (*p* < 0.001) rising from 36.2% in 2011 to 64% in 2019.

With regards to anticoagulation status, 35% (*n* = 1176) of men undergoing surgery were prescribed an oral anticoagulation such as acetylsalicylic acid or coumadin due to co-existing medical conditions. Overall, there was a significant increase of patients on general medication (such as anticoagulation, antidiabetics or pain killers) between 2011 and 2019 (*p* < 0.001).

### Intraoperative baseline results

**Fig. 2:**
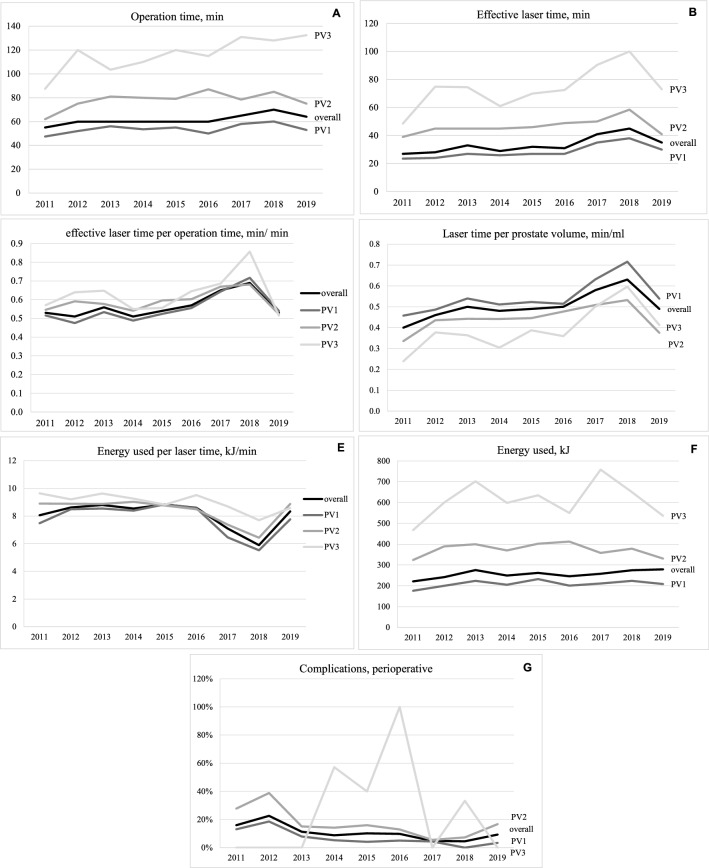
Significant parameters of intraoperative baseline results between 2011 and 2019 in correlation to prostate volume group 1–3, **a** OT, **b** LT, **c** LT/OT ratio, **d** LT/TRUS volume, **e** Energy/LT, **f** Energy used, **g** complications, perioperative

The analysis showed a significant increase in OT (*p* < 0.001) and LT (*p* < 0.001)—see Fig. 2a, b. In subgroup analyses by PV, the OT only showed a significant increase in the PV2 group (81–150 ml) while the LT showed a significant increase in PV1-3. Although the prostate glands in PV3 were significantly larger (up to 330 ml), the LT only increased by approximately 3 min per year (*p* = 0.011), while the LT in PV1 (*p* < 0.001) and PV2 (*p* < 0.001) only increased by approximately 2 min per year.

The applied energy (median 253 kJ, IQR 170–375, PV1 *p* = 0.338, PV2 *p* = 0.725, PV3 *p* = 0.931) stayed constant with no change in energy density (median 3.94 kJ/ml, IQR 2.94–5.02, PV1 *p* = 0.344, PV2 *p* = 0.993, PV3 *p* = 0.192) in the observation period between the PV 1–3 while the energy used (*p* = 0.038) showed a significant increase overall.

The median LT/OT ratio stayed highly efficient over 50% for PV1-3. It was significantly improved between 2011 and 2019 for PV1 (*p* < 0.001) and PV2 (*p* < 0.001) up to over 60% in 2017 and 2018.

Most patients were treated with one fiber (75.6%).

The LT per PV (median 0.5 min/ml, IQR 0.38–0.66) showed a steady increase with 0.025 min/ml (*p* < 0.001) each year. The most obvious increase was observed in PV1 with an increase of 0.029 min/ml each year (*p* < 0.001).

Vice versa the applied laser energy per LT (median 8.45 kJ/min, IQR 6.72–9.44) showed a steady decrease (*p* < 0.001) between 2011 and 2019. Moreover, there was a significant decrease in PV1-3 by approximately 0.3 kJ/min each year as shown in Fig. 2e.

With regard to perioperative AEs, conversion to TURP, which was the most frequent AE observed, decreased from 16% (*n* = 24) in 2011 to 9.4% (*n* = 6) in 2019 (*p* < 0.001) as shown in Fig. 2g. Likewise, perioperative transfusions dropped from 2% in 2011 to 0% in 2019 (*p* = 0.007).

The subanalysis of PV1-3 showed no reduction in intraoperative AEs compared to PV3 (*p* = 0.967).

### Postoperative baseline results

The length of hospital stay remained consistent with a median of two days (*p* = 0.553) and a median postoperative catheterization time of 1 day with a significantly shorter range in PV3 (*p* = 0.043). The need for re-catherization stayed constant across the years (*p* = 0.433, 5.9%).

Overall, the preoperative IPSS/QoL (median 23/4, IQR 19–27/3–5) showed a reduction in symptomatic BPO after 3 months (median 6/1, IQR 4–9 + 1–2, *p* > 0.001) and remained stable at both 6 (median 5/1, IQR 4–9/0–1) and 12 months (median 4/1; IQR 2–7/0–1). Between 2011 and 2019 the preoperative QoL showed a significant decrease by − 0.188 each year (*p* < 0.001). Moreover, there was a significant reduction of QoL after 12 months of surgery (*p* = 0.005).

The median postvoid residual volume was reduced from 120 ml (IQR 30–273) to 30 ml (IQR 0–75, *p* < 0.001) post-operatively, without any changes over the years, and a stable result in our follow-up time up to 12 months after surgery. The median Qmax improved from preoperative 6 ml/s (IQR 4–8) to 3 months postoperative 20 ml/s (IQR 16–22, *p* < 0.001). The outcome stayed stable after 6 and 12 months and between 2011 and 2019.

### Postoperative adverse events

Overall postoperative AEs (Clavien-Dindo classification low-grade and high-grade) were significantly reduced from 2011 with *n* = 106 (48.8%) to 2019 with *n* = 20 (24.7%) (*p* < 0.001). The sub-analysis of PV1-3 showed a significant decrease in postoperative AE especially for prostates from 80 to 150 ml (*p* < 0.001).

**Fig. 3:**
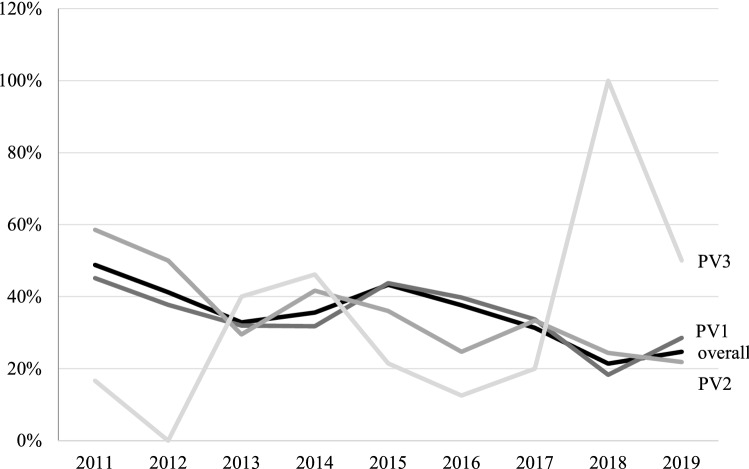
Early postoperative complications (< 30 days) between 2011 and 2019 in correlation to prostate volume group 1–3

The presence of LUTS, which were observed to be the most common postoperative low-grade AE (Clavien-Dindo grade I) decreased from 25% (*n* = 37) in 2011 to 8.8% (*n* = 7) in 2019 (*p* < 0.001) as shown in Fig. 3. Furthermore, postoperative hematuria (14.2–5%, *p* = 0.001) and urinary retention (6.1–0%, *p* = 0.035) significantly decreased. All three of these postoperative AE remained constant across PV1-3.

All other documented complications such as stroke, cardiac ischemia or angina pectoris stayed constant at an extremely low level with a total of 12 cases during the period of 2011–2019.

## Discussion

The present GGG database analysis sought to evaluate the intraoperative progress in photoselective vaporization of the prostate with the GreenLight-XPS 180-Watt system (GL-XPS) in the largest documented cohort (> 3400) treated at seven global high-volume tertiary referral centers. Observing 3441 patients operated by eight surgeons with similar experience in performing this procedure at the start of our study strengthens the observations of intraoperative progress. We demonstrated a temporal improvement in the effectiveness, efficacy, and safety of GL-XPS.

One important aspect of progress with GL-XPS was the significant reduction in intraoperative and postoperative high-grade AEs while operative time and laser time showed a statistically significant increase but stayed highly efficient over the years of surgery and within the prostate PV groups.

First, our OT (> 55 min) and LT (> 27 min) between 2011 and 2019 is in line with data published by the multicenter GOLIATH study with an OT of 49.6 min (mean; SD 21.8) and a LT of 44.5 min (mean, SD 21.5) [6]. However, in comparison to the GOLIATH multicenter study, our LT was more efficient with a median time of 27 min (PV1, IQR 20–38) rising to a median of 75 min (PV3, IQR 54–96) for to the larger glands sized close to 330 ml [10].

More interestingly the LT/OT ratio, as a parameter of intraoperative efficiency stayed significantly high with a value > 50% within each PV group from the beginning of this study and significantly improved during 2011–2019 to > 60%, independent to PV (PV1-3; *p* < 0.001). Our results are in line with multiple previously published results who postulated before, that larger PV prolongs the OT and LT a few minutes but not exponentially [11, 12]. Therefore, it was important to guarantee a high level of energy usage > 200 kJ and an energy density of 4 kJ/ml [10, 13].

Our database showed that the most important prognostic influencing factor for the significantly increasing parameters such as OT, LT, LT/ml or energy density is the PV. Furthermore, it is the most important factor for decreasing parameters such as preoperative and postoperative complications. Valdivieso et al. were able to show that GreenLight-XPS was feasible in men with very large prostates [13]. A more detailed analysis of the impact of the presence of a median lobe was recently published by the GGG showing that the presence of a median lobe has no clinically significant impact on procedural or postoperative outcomes for patients undergoing Greenlight PVP using the XPS-180 W system [14]. The increase in prostate volume and the significant increase in patients with a median lobe can be seen as a signal of the increasing surgical confidence in the Greenlight method for large prostate. 

Progress in safety of GL-XPS is confirmed in our study with significant low intraoperative and postoperative AEs comparable with healthier patients on no permanent anticoagulation. Remaining on anticoagulation with for example acetylsalicylic acid or coumadin (35%, *n* = 1176, *p* = 0.907) while performing GL-XPS, had a low impact on morbidity or high-grade AEs [15].

Perioperative AEs significantly decreased over the years of surgery (*p* < 0.001) and showed in our sub-analysis a significant reduction in prostate glands up to 150 ml (*p* < 0.001) while complication-rate stayed constant on a low level in PV3. These results are in line with Nguyen et al.’s GGG database study which suggested GL-XPS to be safe in larger glands especially those with a median lobe [14] Likewise, Stone et al. and Meskawi et al. further confirmed GL-XPS’ safety for glands > 100 ml [4, 16]. Intraoperative transfusions being one of the most severe low-grade AE significantly (*p* = 0.004) dropped to 0% over time. This value is considered as a very low rate for glands > 100 ml [10].

The postoperative baseline results are in line with multicenter studies. A length of hospital stay with a median of 2 days, high symptom reduction in IPSS/QoL with median of 5/1, sufficient Qmax > 18 ml/s and a median catheterization time of 1 day stayed stable in our study between 2011 and 2019 and showed no difference within the different PV groups [17, 18].

Overall, postoperative AEs were significantly reduced to almost half across the time of our study. More specifically, the most common low-grade AE such as LUTS, hematuria, and urinary retention were reduced significantly between 2011 and 2019, while incontinence stayed constant over the observation period. These results are in line with Goueli et al. who showed significantly different postoperative rates in urinary retention vs. no retention collective comparison. Moreover, our results are confirmed as an extremely low AE rate even in the beginning of our study in 2011. The modest, yet steady reduction of AE rate between 2011 and 2019 further emphasizes the high level of surgeon experience at the start of the study with a potential in progress for each surgeon [19].

Overall, we assume with previous GGG results of Nguyen et al. that the surgeon’s level of experience with the GL-XPS 180-Watt laser system combined with a global experience transfer, together provides an efficient outcome for each high-volume center and a safe operation procedure for each patient [14].

Our GGG analysis is limited by its retrospective design, the short follow-up period of 12 months and that not all parameters were collected for all 3441 patients. Further, detailed parameters such as anticoagulants are incomplete in the GGG data base and therefore could not be analysed. Unfortunately, long term follow-up beyond 12 months is not available on a global scale yet. Each surgeon is already considered to be a highly experienced surgeon in GL-XPS and past their learning curve, operating for years, and may not be representative of an average or minimally experienced GL-XPS surgeon. Despite this limitation, our study is the first analysis to verify the perioperative progress of GL-XPS at a global scale.

## Conclusion

Our GGG analysis showed that, within a 9-year time period, it is possible to significantly decrease intraoperative, transfusions and postoperative adverse events as part of the experience level progress of a surgeon across different prostate volume groups (especially up to 150 ml) while staying highly efficient in the operative time and laser time of GL-XPS.
